# The antifungal protein PAF interferes with PKC/MPK and cAMP/PKA signalling of *Aspergillus nidulans*

**DOI:** 10.1111/j.1365-2958.2009.06936.x

**Published:** 2009-11-19

**Authors:** Ulrike Binder, Christoph Oberparleiter, Vera Meyer, Florentine Marx

**Affiliations:** 1 Biocenter, Division of Molecular Biology, Innsbruck Medical UniversityFritz-Pregl Strasse 3, A-6020 Innsbruck, Austria; 2 Berlin University of Technology, Institute of Biotechnology, Department Microbiology and GeneticsGustav-Meyer-Allee 25, D-13355 Berlin, Germany

## Abstract

The *Penicillium chrysogenum* antifungal protein PAF inhibits polar growth and induces apoptosis in *Aspergillus nidulans*. We report here that two signalling cascades are implicated in its antifungal activity. PAF activates the cAMP/protein kinase A (Pka) signalling cascade. A *pkaA* deletion mutant exhibited reduced sensitivity towards PAF. This was substantiated by the use of pharmacological modulators: PAF aggravated the effect of the activator 8-Br-cAMP and partially relieved the repressive activity of caffeine. Furthermore, the Pkc/mitogen-activated protein kinase (Mpk) signalling cascade mediated basal resistance to PAF, which was independent of the small GTPase RhoA. Non-functional mutations of both genes resulted in hypersensitivity towards PAF. PAF did not increase MpkA phosphorylation or induce enzymes involved in the remodelling of the cell wall, which normally occurs in response to activators of the cell wall integrity pathway. Notably, PAF exposure resulted in actin gene repression and a deregulation of the chitin deposition at hyphal tips of *A. nidulans*, which offers an explanation for the morphological effects evoked by PAF and which could be attributed to the interconnection of the two signalling pathways. Thus, PAF represents an excellent tool to study signalling pathways in this model organism and to define potential fungal targets to develop new antifungals.

## Introduction

Antifungal proteins from filamentous fungi such as PAF and AFP are low molecular weight, cysteine-rich and cationic proteins that inhibit the growth of opportunistic zoo- and plantpathogenic fungi including *A. fumigatus*, *Fusarium graminearum*, *Botrytis cinerea*, and of the model organism *A. nidulans* (reviewed by Marx, 2004; Marx *et al.*, 2008; Meyer, 2008). Although the antifungal activity and some cellular or physiological effects have been studied *in vitro*, only a modest progress has been achieved so far in deciphering their mode of action. For the majority of antimicrobial peptides the disruption of the plasma membrane integrity has been related to their toxic action (Brogden, 2005). However, increasing evidence exists that the species-specific toxicity of many other antimicrobial proteins is mediated by their interaction with distinct molecules or receptors located in the outer layers of the target organisms, e.g. the cell wall or the plasma membrane, from where specific signals are transmitted into the cell (Thevissen *et al.*, 2000; 2004; 2005; Hagen *et al.*, 2007; Marx *et al.*, 2008). Most interestingly, the internalization of some antimicrobial proteins was reported and in few cases even intracellular interaction molecules were identified (Oberparleiter *et al.*, 2003; Moreno *et al.*, 2005; Lobo *et al.*, 2007).

Interference of antifungal toxins with the cell signalling network can have many deleterious effects in target organisms. The cholera toxin, for example, activates the adenylate cyclase by heterotrimeric G-protein signalling causing the hyperactivation of cAMP signalling with fatal effects (Gill and Woolkalis, 1988). Another example, the fungicide fludioxonil, activates the Hog1-type Mpk Osc1, which impairs the function of infection structures of the plant pathogen *Colletotrichum lagenarium* (Kojima *et al.*, 2004). Only recently, it was reported that the plant defensin MsDef1 from *Medicago truncatula* modulates two Mpk signalling cascades in *F. graminearum* (Ramamoorthy *et al.*, 2007).

When microorganisms are exposed to sublethal concentrations of certain toxins, the activation of specific signalling cascades contribute to maintain cellular integrity. One of the best studied response is the fungal cell wall integrity (CWI) pathway. Substances that inhibit fungal growth by interfering with the cell wall biosynthesis, such as Congo red (CR), Calcofluor white (CFW), caffeine, caspofungin or micafungin activate the Mpk and induce the CWI pathway in unicellular and filamentous fungi (Reinoso-Martin *et al.*, 2003; Monge *et al.*, 2006; Fujioka *et al.*, 2007; Meyer *et al.*, 2007; reviewed by Heinisch *et al.*, 1999; Levin, 2005). The master regulator for the CWI pathway is the membrane bound small GTPase Rho that proceeds the signalling to Pkc and Mpk (Guest *et al.*, 2004; Levin, 2005). In the yeast *Saccharomyces cerevisiae*, Mpk signalling results in the activation of the transcription factors Rlm1p and SBF, which control the expression of cell cycle regulated genes and genes involved in cell wall synthesis, reinforcement and remodelling (Igual *et al.*, 1996; Jung, 1999). Congruently, the expression of the α-1,3-glucan synthase genes *agsA* from *A. niger* and *agsB* from *A. nidulans* are induced via RlmA (Damveld, 2005; Damveld *et al.*, 2005; Fujioka *et al.*, 2007). In contrast, the expression of the second *A. nidulans*α-1,3-glucan synthase gene, *agsA* was found to be repressed by RlmA (Fujioka *et al.*, 2007). Furthermore, there is strong evidence that other genes involved in cell wall remodelling are regulated via a MpkA-independent, but so far undefined pathway in *A. nidulans* (Damveld, 2005; Damveld *et al.*, 2005; Fujioka *et al.*, 2007). Thus, the CWI pathway in *A. nidulans* differs from that in *S. cerevisiae*.

*Aspergillus nidulans* cells lacking either RhoA, PkcA or MpkA display pronounced hypersensitivity to cell wall interfering drugs, which can partially be cured by osmotic stabilization (Bussink and Osmani, 1999; Guest *et al.*, 2004; Ichinomiya *et al.*, 2007; Ronen *et al.*, 2007). However, Mpk signalling also controls polar growth, actin microfilament formation, mating, nutrient signalling, calcium signalling and secretion by the cross-talk with other signalling cascades, such as the TOR, Hog or Pka pathways in a variety of fungi (Heinisch *et al.*, 1999; Kojima *et al.*, 2004; Furukawa *et al.*, 2005; Levin, 2005; Munro *et al.*, 2007). Caffeine, for example, does not only activate the CWI pathway, but also negatively interferes with the Ras/cAMP/Pka signalling cascade, as demonstrated in *S. cerevisiae* (Kuranda *et al.*, 2006).

As recently reviewed, PAF provokes complex cellular effects when applied to *A. nidulans* such as hyperbranching, plasma membrane hyperpolarization, increased potassium efflux as well as intracellular accumulation of reactive oxygen species, finally causing apoptosis in *A. nidulans* (Marx *et al.*, 2008). The detrimental effects of PAF are thought to be primarily evoked intracellularly as PAF toxicity is dependent on active internalization of the protein (Oberparleiter *et al.*, 2003). In this study, we aimed at gaining a better insight into the mode of action of the antifungal protein PAF and defining the signalling pathways involved in its toxic effects. Based on the finding that the PAF-orthologous protein AFP from *A. giganteus* activates the CWI pathway (Marx, 2004; Hagen *et al.*, 2007), we investigated the role of the PkcA/MpkA signalling cascade in PAF cytotoxicity. Here we show that the CWI pathway confers basal resistance of *A. nidulans* to PAF. Furthermore, we demonstrate that PAF toxicity is mediated by activation of the Pka signalling cascade. Our findings thus indicate that PAF interferes with at least two distinct signalling pathways, namely cAMP/Pka and Pkc/Mpk signalling.

## Results

### RhoA is not directly involved in PAF toxicity

**Fig. 1:**
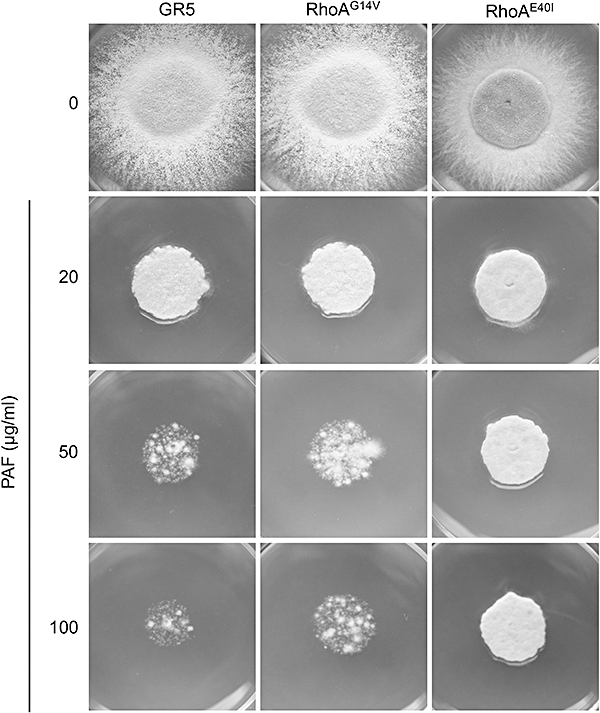
Growth inhibiting effect of 0–100 μg ml^−1^ PAF on the *A. nidulans* RhoA mutant strains, RhoA^G14V^ and RhoA^E40I^, compared with the recipient strain GR5. Controls were left untreated. A total of 2 × 10^3^ conidia were point inoculated on CM agar plates containing appropriate supplements and were incubated for 48 h at 37°C.

It has been shown, that antifungal agents, which inhibit cell wall biosynthesis, induce the CWI pathway by transmission of a respective signal via the membrane bound small GTPase Rho to activate Pkc/Mpk by phosphorylation (Nonaka *et al.*, 1995; Guest *et al.*, 2004; reviewed by Levin, 2005). Importantly, the PAF-related protein AFP from *A. giganteus* was found to bind chitin *in vitro* and, comparable to caspofungin, to increase *agsA* expression, a target gene of the MpkA-activated RlmA transcription factor in *A. niger* (Hagen *et al.*, 2007). To find out, whether PAF evokes a similar cell wall stress response, we tested several *A. nidulans* strains carrying mutations that affect effectors of the CWI pathway. To this end, we investigated the performance of two *A. nidulans* RhoA mutant strains. RhoA is an essential protein in *A. nidulans*. Therefore, *A. nidulans* strains with ectopic copies of the constitutively active *rhoA*^*G14V*^ allele and the dominant *rhoA*^*E40I*^ allele (Guest *et al.*, 2004) were tested for their susceptibility to PAF compared with the recipient strain GR5. Wild-type and mutant strains were incubated with increasing concentrations of PAF (Fig. 1). In agreement with the reported phenotype caused by CFW (Guest *et al.*, 2004), the constitutively active *RhoA*^*G14V*^ strain and the dominant *RhoA*^*E40I*^ strain exhibited a similar sensitivity towards low PAF concentrations (20 μg ml^−1^) as the wild-type. Notably, the dominant *RhoA*^*E40I*^ strain was, however, more resistant to higher PAF concentrations (> 20 μg ml^−1^) than the wild-type or the *RhoA*^*G14V*^ strain which was opposite to the effect of CFW (Fig. S1). This indicates that PAF acts differently on the CWI pathway than the cell wall stressing agent CFW. Furthermore, this result suggests that PAF targets downstream effectors of RhoA. By considering that the dominant *rhoA*^*E40I*^ allele perturbs its GTPase-activating protein (GAP) binding domain and therefore disturbs downstream effectors of Rho-GAP, as proposed by Guest *et al.* (2004), our results revealed that PAF toxicity is not transmitted by RhoA itself, but by RhoA-GAP targets.

### The PkcA/MpkA signalling cascade regulates the sensitivity towards PAF

**Fig. 2:**
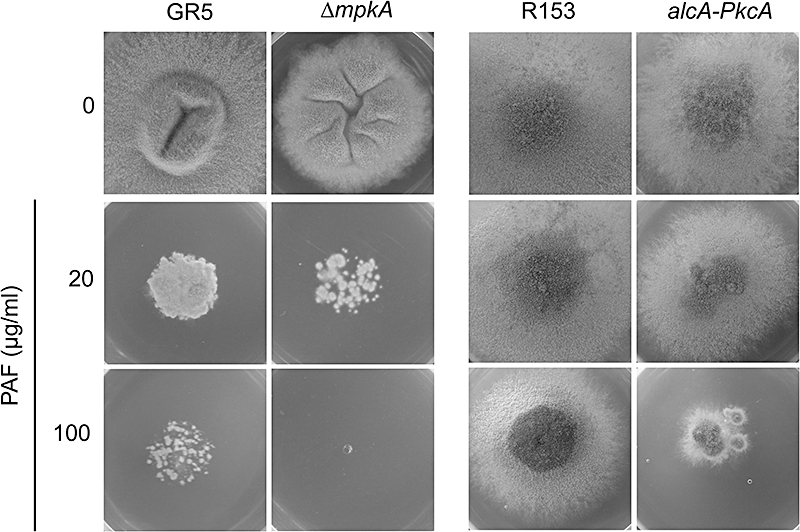
Growth inhibitory effect of PAF on the *A. nidulans* mutants Δ*mpkA* and *alcA-*PkcA compared with the recipient strains GR5 and R153 respectively. A total of 2 × 10^3^ conidia were point inoculated on agar plates (CM for GR5 and Δ*mpkA*, repressive MM containing 1% glucose according to Ronen *et al.*, 2007; for R153 and *alcA-*PkcA) containing the appropriate supplements and 0–100 μg ml^−1^ PAF. The plates were incubated at 37°C for 48 h (GR5 and Δ*mpkA*) or 72 h (R153 and *alcA-*PkcA mutant).

In a next step, we tested an *A. nidulans*Δ*mpkA* mutant strain and a conditional *alcA-*PkcA mutant for their susceptibility towards PAF. In the latter mutant, *pkcA* expression is repressed when grown on glucose. Cell wall stressing agents increase the phosphorylation of the Pkc and the Mpk in fungi, e.g. in the yeast *S. cerevisiae* and *A. nidulans* (Levin, 2005; Fujioka *et al.*, 2007). In consequence, a hypersensitivity of the respective deletion (Δ*mpkA*) or conditional mutant (*alcA-*PkcA) strains of *A. nidulans* to such agents was reported (Bussink and Osmani, 1999; Ichinomiya *et al.*, 2007; Ronen *et al.*, 2007). When exposed to PAF, both *A. nidulans* mutant strains exhibited a hypersensitive phenotype compared with the respective wild-type strains GR5 and R153 (Fig. 2). This phenotype resembled those under treatment with the cell wall modulating agents CFW and caffeine (Figs S1 and S2). By immunoblotting experiments, we further investigated whether PAF increases the phosphorylation of MpkA in analogy to the mode of action of caffeine and CFW (Levin, 2005; Kuranda *et al.*, 2006). Whereas an increase in the phosphorylation of MpkA was evident after a 90 min exposure to 20 μg ml^−1^ CFW compared with the untreated control, we could not detect any significant increase in MpkA phosphorylation after 30 or 90 min of exposure to 50 μg ml^−1^ PAF (Fig. S3), suggesting that PAF fails to activate MpkA. Notably, additional phosphorylated lower molecular weight bands were detectable which were more prominent in the PAF-treated samples. These bands could represent MpkA degradation products arising from PAF treatment. Considering the hypersensitive phenotypes of both *alcA*-PkcA and Δ*mpkA* mutant strains as well as the observation that exposure to PAF does not result in increased MpkA phosphorylation, could hint at the possiblitity that PAF also fails to activate PkcA. However, this speculation awaits further investigations.

### PAF does not activate the CWI pathway

Since PAF interfered with the PkcA/MpkA signalling in a different way than cell wall stressing agents, we wanted to prove that PAF is not activating the CWI pathway. Therefore, we tested the effect of PAF on downstream targets of the CWI pathway, namely genes involved in cell wall biosynthesis and remodelling. A central regulator of CWI is the MADS-box transcription factor RlmA in *A. niger* and Rlm1p in *S. cerevisiae* (Damveld *et al.*, 2005; Fujioka *et al.*, 2007). Northern blot analysis revealed that the expression of the *A. niger rlmA* orthologue in *A. nidulans* was not influenced by 50 μg ml^−1^ PAF (Fig. S4), although a minor decrease in *rlmA* expression level cannot be ruled out. Likewise, the expression of one of the primary targets of *A. nidulans* RlmA, namely *agsB*, was not significantly enhanced in response to PAF treatment. Other genes involved in cell wall synthesis, such as genes encoding chitin synthases (*chsA*, *chsB*, *chsD*, *csmA*), chitinase A (*chiA*) and β-1,3-glucan synthase (*fksA*) were tested for their expression patterns. No main difference in their expression activity could be observed (Fig. S4). However, a slight reduction in *chsA* (−15%) and *chsB* (−10%) expression in response to PAF was detectable by the quantification of the mRNA signals compared with the expression in the untreated controls. This was also reflected by the fact that after a 3 h exposure to PAF, the chitin content was only minimally reduced to 84 ± 4% compared with that of untreated controls. Further evidence that PAF does not induce expression of CWI target genes, resulted from lacking significant nuclear fluorescence in the transgenic *A. niger* strain RD6.47, carrying a nuclear-targeted GFP protein fused to the *A. niger agsA* promoter (Fig. S5).

Assuming that CFW activates the Pkc/Mpk signalling cascade, whereas PAF fails activation, we hypothesized that both compounds combined would either antagonize each other or have no significantly aggravating effects on the growth of *A. nidulans*. We thus quantified the growth rates by determining the colony diameters of surface cultures subjected to 100 μg ml^−1^ PAF and/or 100 μg ml^−1^ CFW. Indeed, when both compounds were applied together no major deterioration of the growth rate of the wild-type strain FGSC4A was observed (47% and 30% growth in the presence of CFW and PAF, respectively, compared with 39% growth in the presence of both compounds; Table 1). Actually, a very modest melioration of growth could be observed when *A. nidulans* was treated with a combination of both compounds compared with the growth rate in the presence of PAF alone (39% versus 30% growth).

**Table 1:** The growth of *A. nidulans* FGSC4A in response to treatment with PAF (100 μg ml^−1^), caffeine (20 mM), CFW (100 μg ml^−1^) or a combination of PAF plus caffeine or PAF plus CFW after 72 h of incubation at 37°C.

Compound	Colony diameter (mm)	% growth
Untreated	20.0 ± 0.0	100
PAF	6.0 ± 0.7	30
Caffeine	6.4 ± 0.5	32
PAF + caffeine	9.0 ± 0.0	45
CFW	9.4 ± 0.6	47
PAF + CFW	7.8 ± 0.8	39

The % of growth was calculated from % changes in radial growth of compound-treated samples compared with untreated controls (= 100%). Colony diameters are given as mean ± SD (*n* = 5).

### cAMP/Pka signalling mediates PAF toxicity in *A. nidulans*

**Fig. 3:**
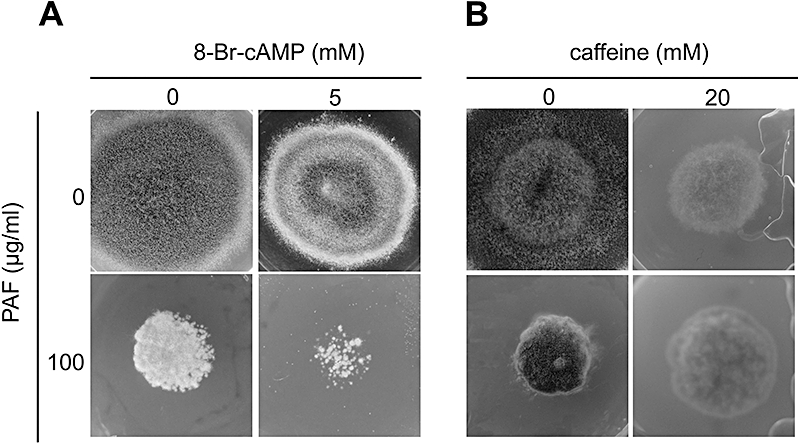
Effect of the cAMP/PkaA signalling pathway modulating substances (A) 8-Br-cAMP and (B) caffeine in combination with PAF on the growth of *A. nidulans* (FGSC4A). A total of 2 × 10^3^ conidia were point inoculated on CM agar plates containing 100 μg ml^−1^ PAF together with 5 mM 8-Br-cAMP and 20 mM caffeine. Plates were incubated at 37°C for 48 h in (A) and 72 h in (B).

**Fig. 4:**
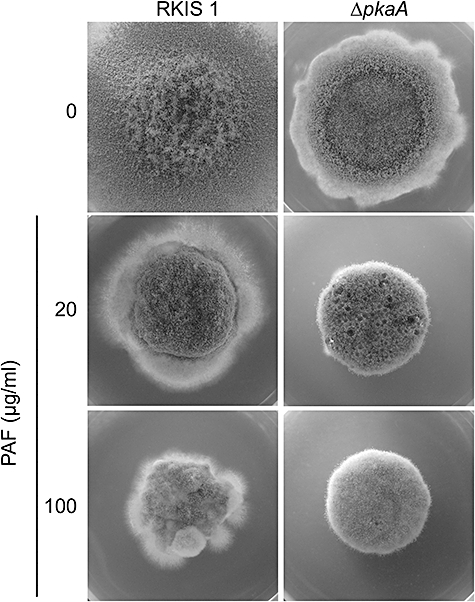
Growth inhibiting effect of PAF on the *A. nidulans*Δ*pkaA* mutant in comparison to the respective recipient strain RKIS1. A total of 2 × 10^3^ conidia were point inoculated on CM agar plates containing the respective supplements and incubated for 72 h at 37°C.

**Fig. 5:**
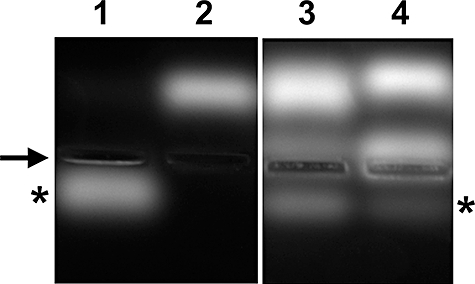
Detection of cAMP-dependent PKA phosphorylation activity. Two micrograms of PepTaq A1 peptide were incubated with 50 μg of crude protein extract in a final volume of 25 μl for 30 min as described in the manufacturer's instructions. Samples were separated on a 0.8% agarose gel at 100 V for 20 min. Lanes 1–4: (1) phosphorylated PepTag A1 peptide (positive control) (2) unphosphorylated PepTag A1 peptide (negative control) (3) PepTag A1 peptide exposed to crude cell extract from *A. nidulans* FGSC4A treated with 100 μg ml^−1^ PAF for 90 min and (4) PepTag A1 peptide exposed to crude cell extract from untreated *A. nidulans*. The arrow indicates the loading wells of the agarose gel, and the asterisks indicate the phosphorylated PepTag A1 peptide, which migrated towards the cathode.

In the course of defining signalling pathways involved in PAF toxicity, we first tested in a pharmacological approach substances that modulate the cAMP/Pka signalling cascade. This signalling pathway and its central protein Pka is a regulator of stress response in many eukaryotes (Zhao *et al.*, 2006). *A. nidulans* was grown in the presence of PAF and 8-Br-cAMP, an activator of fungal cAMP-Pka signalling (Gorovits and Yarden, 2003). PAF aggravated the toxicity of 8-Br-cAMP in *A. nidulans* as shown in Fig. 3A, suggesting that PAF activates the cAMP/Pka signalling cascade. In contrast to 8-Br-cAMP, caffeine reduces the cAMP level and consequently downregulates the Pka signalling pathway in *S. cerevisiae* which is also associated with growth reduction (Kuranda *et al.*, 2006). This holds true for *A. nidulans* FGSC4A as well (Fig. 3B). In the presence of 20 mM caffeine, growth reached only 32% of the untreated control, which was set to 100% (Table 1). In samples treated with 100 μg ml^−1^ PAF, the growth rate was only 30% compared with the untreated control. Notably, a combination of PAF and caffeine moderately ameliorated growth (45% growth compared with the untreated control), rather than reducing it further (Fig. 3B, Table 1), suggesting that PAF and caffeine act in an opposite manner on Pka signalling. However, it has to be noted that PAF was not able to restore the caffeine-specific delay in conidiation (Fig. S6). To prove the specificity of this pharmacological approach, we tested a *pkaA* deletion mutant (Shimizu and Keller, 2001) for its susceptibility towards PAF and used caffeine as a control drug. As shown in Fig. 4 and Table 2, deletion of the *pkaA* gene rendered *A. nidulans* less sensitive to PAF. In the presence of 100 μg ml^−1^ PAF, the growth of the Δ*pkaA* strain was 59%, whereas the isogenic recipient strain RKIS1 reached only 22% growth when compared with the respective untreated controls (100%). The results presented in Fig. S2 and Table 2 provided evidence that the *pkaA* deletion strain exhibits hypersensitivity towards caffeine. Importantly, this caffeine-dependent phenotype of the Δ*pkaA* mutant was not fully curable by PAF addition (Table 2). These results parallel well with growth inhibition assays performed in liquid medium (Fig. S7). Further evidence to our hypothesis that PAF toxicity is mediated at least in part by the activation of the cAMP/PkaA signalling cascade was gained by a biochemical approach. We could prove a moderate, but significant activation of PkaA in crude cellular extracts of *A. nidulans* treated with 100 μg ml^−1^ PAF for 90 min compared with the untreated control. The increase in the amount of phosphorylated PepTag A1 peptide – a highly specific PKA substrate – was evident by a stronger fluorescent signal migrating towards the cathode (Fig. 5). Notably, the separation of the unphosphorylated portion of the PepTag A1 peptide towards the anode resulted in two differently migrating signals (Fig. 5, lanes 3 and 4). A possible explanation for this observation could be that crude cell extracts from *A. nidulans* were used in these samples, whereas purified, active PKA from bovine heart was used for the positive and negative controls (Fig. 5, lanes 1 and 2) as suggested by the manufacturer's instructions. The crude cell extracts might contain factors that interfere with the migration of the PKA substrate peptide.

**Table 2:** The growth of *A. nidulans*Δ*pka* and its respective isognenic control strain RKIS1 in response to treatment with PAF (20 and 100 μg ml^−1^), caffeine (10 and 20 mM), or a combination of PAF plus caffeine after 48 h of incubation at 37°C.

	RKIS1		Δ*pkaA*	
Compound	Colony diameter (mm)	% growth	Colony diameter (mm)	% growth
Untreated	18.3 ± 0.6	100	10.7 ± 0.6	100
Caffeine 10	9.0 ± 0.0	49	5.0 ± 0.0	47
Caffeine 20	5.0 ± 0.0	27	No growth	0
PAF 20	7.7 ± 0.6	42	7.3 ± 0.6	69
PAF 100	4.0 ± 0.0	22	6.3 ± 0.6	59
P 20 + caf 10	9.7 ± 0.6	53	5.0 ± 0.0	47
P 100 + caf 10	7.0 ± 0.0	38	5.0 ± 0.0	47

The % of growth was calculated from % changes in radial growth of compound-treated samples compared with untreated controls (=100%). Colony diameters are given as mean ± SD (*n* = 5).

Taken together, our observations indicated that (i) PAF toxicity is mediated at least in part by the activation of the cAMP/PkaA signalling cascade and (ii) PAF exhibits an opposite effect on the growth of the Δ*pkaA* strain than caffeine. (iii) Finally, the *pkaA* deletion mutant still exhibited caffeine sensitivity. This suggested that caffeine might not only interfere with PkaA, but also with other signalling compounds as well, for example with the secondary Pka catalytic subunit of *A. nidulans*, PkaB (Ni *et al.*, 2005).

### Actin expression and chitin deposition in the cell wall are disturbed under PAF exposure

**Fig. 6:**
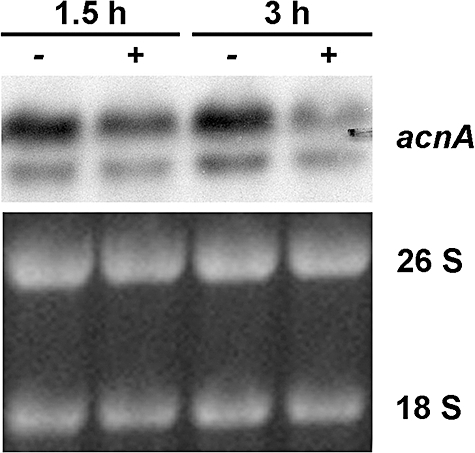
Northern blot analysis of actin (*acnA*) gene expression. Sixteen-hour-old *A. nidulans* cultures were exposed to 0 (−) and 50 μg ml^−1^ PAF (+) for 1.5 and 3 h respectively. Ten micrograms of total RNA was detected for actin gene expression by hybridizing the blotted samples with an actin-specific DIG-dUTP probe (upper panel). The lower panel shows the 26S and 18S rRNA stained with ethidium bromide as loading control. Note that expression of the *A. nidulans acnA* gene results in two transcripts due to the presence of different 3′ termini (Fidel *et al.*, 1988).

**Fig. 7:**
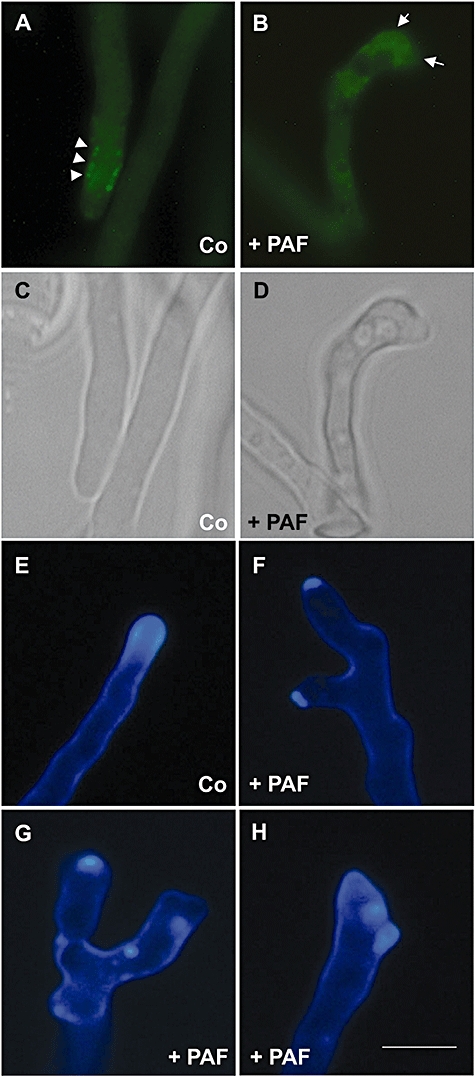
Fluorescence micrographs showing actin and chitin distribution in *A. nidulans* hyphae in response to PAF treatment (+PAF) (B, D and F–H) compared with untreated controls (Co) (A, C and E). (A–D) shows the transgenic *A. nidulans* strain expressing actin-GFP (Taheri-Talesh *et al.*, 2008). (A) In the untreated control, characteristic actin patches at the subapical region of the hyphal tip are visible (white arrow heads). (B) When exposed to 50 μg ml^−1^ PAF, no actin patches were detectable and transition from polar to apolar growth became evident (white arrows). (C and D) Light micrographs of (A) and (B) respectively. (E–H) shows CFW staining of *A. nidulans* FGSC4A. (E) The untreated control sample exhibits a characteristic cap-like CFW fluorescence at the hyphal tip which corresponds to the site of chitin assembly. (F–H) Incubation with 50 μg ml^−1^ PAF induces hyperbranching (F) a reduced chitin content and (G and H) a delocalized chitin deposition at the hyphal tips. Scale bar, 10 μm.

Increase in PkaA activity has been reported to directly or indirectly affect polarized growth of *A. niger* by causing a polar–apolar transition in hyphal morphology (Bencina *et al.*, 2005), an observation that resembles the morphological changes of *A. nidulans* exposed to PAF (Kaiserer *et al.*, 2003). As the polar growth mode of *A. nidulans* is also dependent on polarized actin and tip-localized chitin synthesis, we studied the effect of PAF on actin and chitin localization in *A. nidulans*. Most interestingly, we found that actin (*acnA*) transcription was slightly but significantly repressed after 90 and 180 min of treatment with 50 μg ml^−1^ PAF (Fig. 6). The quantification of mRNA signals in response to PAF treatment revealed an *acnA* expression of 87% and 65% in the samples exposed for 90 and 180 min, respectively, compared with the expression in untreated controls (= 100%). This shortage in actin was equally reflected by a decreased fluorescent signal in PAF-exposed transgenic *A. nidulans* hyphae expressing actin-GFP (Taheri-Talesh *et al.*, 2008) (Fig. 7A–D). In addition, typical actin patches as usually present at the hyphal tips of untreated hyphae were lacking in hyphae treated with 50 μg ml^−1^ PAF (Fig. 7A and B), indicating that actin polarization and/or localization is disturbed in the presence of PAF. Since intact actin filament formation is a prerequisite for polar growth and deposition of chitin in the cell wall, we studied the chitin distribution at the tips of PAF exposed hyphae by CFW staining. Both PAF-treated samples and untreated controls showed an even distribution of chitin in the subapical cell wall and septa (Fig. 7E–H). In untreated hyphae, however, the chitin was cap-like distributed at the hyphal tips, where most of the newly synthesized chitin is deposited (Fig. 7E). In contrast, a reduction in chitin content accompanied by a chitin misdistribution at the tips could be observed in PAF-treated hyphae (Fig. 7F–H).

## Discussion

**Fig. 8:**
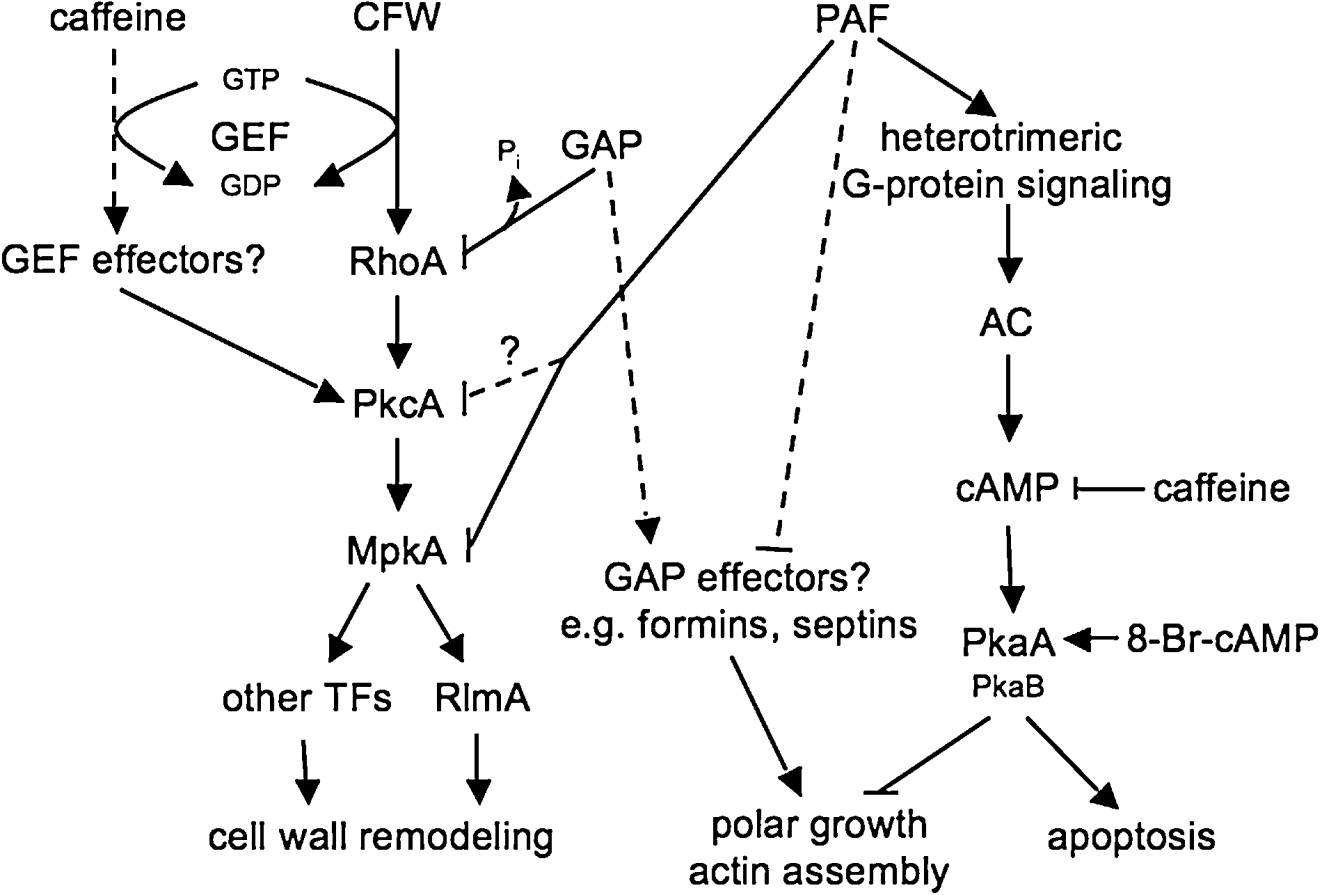
Tentative model for the mode of action of the antifungal protein PAF in *A. nidulans*. PAF activates the cAMP/PkaA signalling cascade via heterotrimeric G-protein signalling (Leiter *et al.*, 2005), which leads to apoptosis and to defective actin polymerization and apolar growth (this work and Leiter *et al.*, 2005). Note that the secondary catalytic subunit PkaB could be involved in signal transmission of PAF and caffeine due to partly redundant functions in PkaA signalling in *A. nidulans* (Ni *et al.*, 2005). Actin assembly and polar growth might also be disturbed via so far unidentified RhoA-GAP effectors. PAF further fails to activate MpkA and probably also PkcA (this work), disrupting basal resistance of *A. nidulans* towards antifungal activity. In contrast, the cell wall interfering drugs CFW and caffeine induce CWI signalling and cell wall remodelling in a RhoA-dependent and RhoA-independent way respectively (this work and Kuranda *et al.*, 2006; Fujioka *et al.*, 2007). The cell wall remodelling by MpkA-independent pathways, as documented for *A. nidulans* (Fujioka *et al.*, 2007), were not considered in this diagram. Dotted lines and question marks represent so far unidentified connections. AC, adenylate cyclase; CFW, Calcofluor white; GAP, GTPase activation protein; GEF, guanine nucleotide exchange factor; PAF, *Penicillium chrysogenum* antifungal protein; TFs, transcription factors.

With this study, we have initiated the investigation of the pathways involved in the transmission of signals related to the exposure to the antifungal protein PAF in the genetic model organism *A. nidulans*. We tested whether RhoA, the major stress signal processing protein of the CWI pathway, plays a central role in mediating a PAF-specific signal to the downstream effectors PkcA and MpkA. Similarly to the phenotypes evoked by CFW, the wild-type strain and the constitutively active RhoA^G14V^ mutant were likewise sensitive to PAF. However, the dominant *rhoA*^*E40I*^ allele in *A. nidulans* conferred reduced sensitivity to PAF, which stands in contrast to the hypersensitive phenotype induced by CFW. The RhoA^E40I^ mutant grows with hyperbranching compared with the wild-type. This mutation affects the effector domain of RhoA and perturbs the GAP binding domain (Saka *et al.*, 2001; Guest *et al.*, 2004). It was hypothesized that this dominant mutation disturbs downstream targets of GAP, which have not been identified so far in *A. nidulans*. The increased resistance of the RhoA^E40I^ mutant at PAF concentrations > 20 μg ml^−1^ could reflect a GAP target-dependent survival mechanism that might be responsive only at higher PAF concentrations. Putative candidates for downstream targets of GAP could be for example the formin SepA and the septin AspB as suggested by Guest *et al.* (2004). Formins are a family of scaffold proteins that integrate both signalling proteins and actin binding proteins and regulate actin assembly, septum formation and polarized growth. Furthermore, SepA is required for actin ring formation in *A. nidulans* hyphae (Sharpless and Harris, 2002). The septin AspB is involved in the emergence of secondary germ tubes (Westfall and Momany, 2002). These were affected in the dominant Rho^E40I^ mutant. It was thus hypothesized elsewhere that proper function of SepA and AspB depends on RhoA activity (Guest *et al.*, 2004). Therefore, the PAF-resistant phenotype of the Rho^E40I^ and the transition from polar to apolar growth in the presence of PAF could indicate a link between PAF activity, downstream targets of RhoA and actin regulation, apart from or even in addition to the involvement of cAMP/Pka signalling (Fig. 8).

In contrast to well known CWI pathway inducers, such as CFW and caffeine, no increase in MpkA phosphorylation could be determined in response to PAF. If PAF exerts its toxicity by failing or reducing the Pkc/Mpk pathway activation, the repression of *pkcA* or deletion of the *mpkA* gene would enforce its activity and result in hypersensitive phenotypes. Indeed, we observed hypersensitivity of the respective mutant strains towards PAF. Moreover, the combination of CFW with PAF did not aggravate CFW-induced growth inhibition of *A. nidulans*, which further suggested that PAF does not activate the CWI pathway. These results enable us to conclude that PAF evokes other effects not mainly related to the remodelling of the cell wall as discussed in the following.

Our study demonstrates that PkcA/MpkA signalling confers a basal resistance to PAF-treated *A. nidulans* cells. However, PAF had no significant effect on the transcription levels of cell wall remodelling enzymes. Therefore, we conclude that PAF is not primarily a cell wall stressing drug. This hypothesis is supported by the fact that osmotic stabilizers are not able to cure the cytotoxic effect of PAF (data not shown) and that PAF does not possess any chitin binding activity *in vitro* (Batta *et al.*, 2009). Furthermore, the *agsA*-GFP reporter system in *A. niger* did not respond to PAF exposure. This stands in contrast to the effects provoked by the PAF-related AFP. The AFP-specific cell wall stress is reflected by a significant decrease in chitin synthase activity and the induction of the *agsA* promoter in *A. niger* (Hagen *et al.*, 2007). A further indication for a cell wall-independent activity of PAF is the induction of a programmed cell death (PCD) phenotype in PAF-treated *A. nidulans* protoplasts (Leiter *et al.*, 2005). Therefore, we conclude that, in contrast to AFP, the cell wall of *A. nidulans* seems not to be imperative for the antifungal activity of PAF. However, it cannot be excluded that hypersensitivity of both mutant strains, Δ*mpkA* and *alcA-PkaA*, towards PAF arises from a modified cell wall composition which in consequence facilitates the access of the antifungal protein to the cell.

In any case, PAF and AFP definitely show up diverged modes of action, a phenomenon that is well documented for other closely related antimicrobial proteins, e.g. for the antifungal plant defensins MsDef1 and MtDef4 from *Medicago* spp. (Ramamoorthy *et al.*, 2007).

This study furthermore showed that PAF antagonizes caffeine toxicity. Caffeine elicits pleiotropic effects leading to cell death by a so far uncharacterized mechanism. Recent investigations in *S. cerevisiae* elucidated its potency as cell wall interfering agent that has been shown to modulate more than one signalling pathway. It reduces the cAMP level and activates Pkc1p/Mpk1p signalling by inhibition of the Tor1 kinase (Kuranda *et al.*, 2006). In this respect, mutants defective in Pkc1p/Mpk1p are hypersensitive to caffeine. Activation of a cell wall remodelling response indicated that the CWI pathway is induced by caffeine. Notably, the cell wall modifications caused by this drug were independent of the transcriptional activation of Rlm1p-regulated genes, giving evidence that the Pkc/Mpk cascade is not only activated by cell surface sensors at the ‘top’ but can be laterally induced as well (Harrison *et al.*, 2004).

No information of the caffeine effect on the signal transduction in *A. nidulans* is available so far and our investigations are the first to confirm hypersensitivity of the *A. nidulans alcA*-PkcA and Δ*mpkA* strains to caffeine. This demonstrates that the activation of the PkcA/MpkA signalling cascade by this compound in *A. nidulans* is similar to the situation found in *S. cerevisiae*. However, the phenotypes of the constitutively active allele *rhoA*^*G14V*^ and the dominant allele *rhoA*^*E40I*^ resembled the *A. nidulans* wild-type strain. This indicates that RhoA is not directly involved in the transduction of the caffeine-specific signal. This contrasts to the situation present in *S. cerevisiae*. In this organism, the Rho1p GTP/GDP exchange factor Rom2p was identified to transduce the caffeine-related response by modulating the Pkc1p/Mpk1p activation and the intracellular cAMP level, but no evidence was given so far that proves a direct involvement of Rho1p in the signal transduction (Park *et al.*, 2005; Kuranda *et al.*, 2006). Thus it might be possible that Rom2p acts via downstream effectors other than Rho1p. This hypothesis, if valid also for *A. nidulans*, could explain the RhoA-independent caffeine signal transduction.

Caffeine was shown to reduce the cAMP level in *S. cerevisiae*, thus negatively interfering with Pka signalling (Kuranda *et al.*, 2006). It has to be noted, that this mechanism is opposite to that found in mammalian cells, where caffeine increases the cAMP level by inhibition of the enzyme phosphodiesterase (Butcher and Potter, 1972). Notably, in the *A. nidulans*Δ*pka* mutant, caffeine induced a hypersensitive phenotype, which implies the involvement of another signalling compound affected by caffeine, other than PkaA. This could be, for example, the secondary Pka catalytic subunit, PkaB, which shows partly overlapping functions with PkaA (Ni *et al.*, 2005). This assumption is supported by the observation that yeast strains with low Pka activity are more sensitive towards caffeine than the wild-type controls (Kuranda *et al.*, 2006). However, it cannot be ruled out that caffeine interferes with other, so far uncharacterized, signalling pathways involved in the transmission of its pleiotropic effects and which contribute to the reduction of the survival rate of the *A. nidulans*Δ*pka* mutant.

Since PAF slightly relieved the caffeine-specific growth inhibition in *A. nidulans* wild-type, we propose that the activation of the cAMP/PkaA signalling cascade accounts at least in part for PAF toxicity. This conclusion is emphasized by the fact that the Δ*pkaA* mutant exhibited a reduced PAF-sensitivity than the isogenic control strain RKIS1 and the wild-type strain FGSC4A. This result was further consolidated by the finding that the PkaA activity of PAF-treated cells appeared higher than in the control samples. Furthermore, PAF aggravated the toxicity of the pharmacological inducer of the cAMP/PkaA signalling pathway, 8-Br-cAMP, in *A. nidulans* FGSC4A and did not further deteriorate the caffeine activity in an additive or synergistic way in the wild-type or mutant strains tested. On the contrary, caffeine moderately ameliorated at distinct concentrations the PAF-dependent growth inhibition. Notably, PAF was not able to cure the delay in asexual development of caffeine-treated samples. Taking into account the numerous uncharacterized pleiotropic effects of caffeine beside the involvement in Pkc/Mpk and cAMP/Pka signalling, it can be assumed that PAF is interfering with the growth inhibitory effect of caffeine, but is apparently not affecting the developmental delay which might be regulated by other pathways.

The components of the cAMP/PkaA signalling pathway are well conserved among eukaryotes (Francis and Cobrin, 1994; van Biesen *et al.*, 1996; Fillinger *et al.*, 2002) and the cascade has been shown to be involved in a number of developmental events. In fungi, the activation of the cAMP/Pka pathway has not only been linked to small G-proteins, like Ras2p, but to heterotrimeric G-proteins as well (Kronstad *et al.*, 1998; Thevelein and de Winde, 1999; Shimizu and Keller, 2001). In a previous study, we have demonstrated that heterotrimeric G-protein signalling is involved in PAF toxicity. The dominant interfering *fadA*^*G203R*^ allele conferred less sensitivity to PAF in *A. nidulans*. Thus, the active heterotrimeric G-protein signalling is necessary for processing a PAF-specific signal (Leiter *et al.*, 2005). According to Shimizu and Keller (2001), the phenotype of a Δ*pkaA* mutant resembles the *fadA*^*G203R*^ mutant phenotype, which strengthens our findings (Shimizu and Keller, 2001).

There are several reports showing that heterotrimeric G-protein signalling and the cAMP/Pka signalling cascade can modulate PCD. Semighini *et al.* (2006) observed that the heterotrimeric G-protein complex is part of the signal transduction pathway that promotes apoptosis in *A. nidulans* exposed to farnesol. This corroborates our assumption that PAF triggers PCD via heterotrimeric G-protein signalling (Leiter *et al.*, 2005) and further substantiate our present finding that this effect might be mediated by the activation of the cAMP/Pka signalling cascade (Fig. 8).

We observed a reduction of the chitin content and a delocalization of chitin at the tips of PAF-treated hyphae. This observation explains well the PAF-dependent morphological alterations reported, e.g. hyperbranching and depolarized growth (Kaiserer *et al.*, 2003). In parallel, the decrease in *acnA* gene expression indicated a shortage of this cytoskeleton-related compound in PAF-treated cells. This was further underlined by a significant reduction of actin-specific fluorescent signals at the hyphal tips, the place of intense growth metabolism, secretion of extracellular enzymes, and the delivery of cell wall and membrane synthesis material. Reduced actin expression might thus account for the disturbance of actin polarization and a severe deregulation of chitin delivery to the tips. However, at this point it remains unclear whether these observations can be assigned to a primary or secondary effect of PAF toxicity on *A. nidulans*. Microarray analysis is currently in progress to obtain an overview on deregulated genes in response to PAF-treatment, which will provide more information about potential targets of PAF and signalling pathways involved in PAF toxicity.

### Conclusion

As summarized in Fig. 8, we propose that the activity of PAF is modulated by at least two signalling pathways. This finding significantly contributes to explain some features of the mode of action of PAF, which were identified so far, for example changes in morphology, heterotrimeric G-protein signalling and PCD. PAF activates the cAMP/Pka signalling cascade, possibly via heterotrimeric G-protein signalling. This could explain the induction of apoptosis in PAF-treated fungal cells as reported in our previous study (Leiter *et al.*, 2005), and could account for the polar–apolar growth transition. In contrast, Pkc/Mpk signalling mediates basal resistance to PAF. The removal of the basal resistance by disturbing the function of PkcA and MpkA (as it is present in the *alcA-PkcA* and the Δ*mpkA* mutants) aggravates the toxicity of PAF. Based on the fact that PAF is not activating the CWI pathway, PAF-dependent cell wall remodelling can be excluded. Since the cAMP/Pka and the Pkc/Mpk signalling pathways control numerous cellular events, the interference of PAF with these pathways could account for its detrimental effects on target organisms. Further examinations will be necessary to unravel the question whether a cross-talk with additional signalling cascades exists and how these pathways are interconnected. In this respect, PAF represents an excellent tool to disclose the so far unknown interrelation(s) between cAMP/Pka and the Pkc/Mpk signalling in *A. nidulans*. Importantly, the dissection of the signalling pathways involved in PAF toxicity will also contribute to the identification of potential fungal targets for the development of new strategies to combat fungal infections.

## Experimental procedures

### Strains, media and chemicals

Fungal strains used in this study are listed in Table 3. Except for the *A. nidulans* strains R153 and *alcA-*PkcA, all fungi were grown in complete medium (CM) (Kuranda *et al.*, 2006) with the respective supplements where needed (Bussink and Osmani, 1999; Shimizu and Keller, 2001; Guest *et al.*, 2004). R153 and *alcA-*PkcA were grown in defined minimal medium (MM) according to Ronen *et al.* (2007). Unless otherwise stated, all chemicals were purchased from Sigma (Vienna, Austria).

**Table 3:** Fungal strains used in this study.

Strains	Relevant genotype	Source or reference
*A. nidulans*		
FGSC 4A	Glasgow wild-type	FGSC
R153	*wA2; pyroA4*	Ronen *et al.* (2007)
*alcA*-PkcA	*wA2; pyroA4; pyrG89::pyr4alcA(p)::pkcA*Δ*p*	Ronen *et al.* (2007)
GR5	*pyrG89; wA3; pyroA4*	Guest *et al.* (2004)
RhoA^G14V^	A773 + pGG2 (*rhoA*^G14^) and pRG3AMA1 (co-transformation plasmid)	Guest *et al.* (2004)
RhoA^E401^	A773 + pGG5 (*rhoA*^E401^) and pRG3AMA1 (co-transformation plasmid)	Guest *et al.* (2004)
GR5	*wA3; pyroA4; pyrG89*	Bussink and Osmani (1999)
Δ*mpkA*	Δ*mpkA*	Bussink and Osmani (1999)
RKIS 1	*papaA1; yA2*	Shimizu and Keller (2001)
Δ*pkaA*	*papaA1; yA2;*Δ*pkaA::argB;*Δ*argB::trpC; trpC801; veA1*	Shimizu and Keller (2001)
Actin GFP	*wA3; pyroA4; actin_GFP::pyrG (pyr G 89)*	Taheri-Talesh *et al.* (2008)
*A. niger*		
RD6.47	P*agsA::h2b::egfp::*T*trpc*	Meyer *et al.* (2007)

### Purification of PAF

PAF was purified from the supernatant of 72 h cultures of *Penicillium chrysogenum* Q176 (ATTC 10002). The supernatant was cleared by centrifugation and ultrafiltration and then loaded on a CM-sepharose column as described previously (Kaiserer *et al.*, 2003). Eluted fractions containing PAF were pooled, dialysed against phosphate buffer (10 mM Na-phosphate, 25 mM NaCl, pH 6.6), concentrated and filter sterilized. The protein concentration was determined photometrically and by SDS-PAGE.

### Growth inhibition assays

To determine fungal growth, 2 × 10^3^ conidia of the respective *A. nidulans* strains were spotted in 5 μl aliquots on appropriate solid media and – where needed – supplemented with increasing concentrations of various growth affecting compunds (PAF, caffeine, CFW, 8-Br-cAMP). The plates were then incubated at 37°C for up to 72 h. Every 24 h, the plates were photographed and the colony diameters determined. The percentage (%) of growth was calculated from % changes in radial growth of compound-treated samples compared with untreated controls (=100%). Plate assays were set up in duplicates and repeated at least twice. Liquid growth inhibition assays were performed in 96-well plates as described previously (Kaiserer *et al.*, 2003).

### Determination of the chitin content

The chitin content of PAF-treated versus untreated *A. nidulans* hyphae was determined according to the amount of glucosamine liberated from the cell wall of lyophilized mycelium by H_2_SO_4_ hydrolysis as described by Soulie *et al.* (2003).

### Staining of chitin with CFW

Chitin distribution in the cell wall of *A. nidulans* hyphae was investigated with the fluorescent stain CFW (Fluorescent brightener 28 free acid, Sigma). *A. nidulans* conidia were germinated on glass slides for 16 h before hyphae were treated with 50 μg ml^−1^ PAF at RT for 3–6 h. CFW was applied in a final concentration of 11 μM (diluted in CM) for 8 min in darkness. Stained samples were mounted and visualized with a Zeiss Axioplan fluorescence microscope (excitation filter G 365, emission filter LP 420) equipped with a Zeiss AxioCam MCr digital camera.

### Analysis of actin distribution in *A. nidulans*

To visualize the impact of PAF on the distribution of actin in hyphal tips, an *A. nidulans* GR5 strain was used that carries an additional copy of actin tagged with GFP on its N-terminus (Taheri-Talesh *et al.*, 2008). The strain was grown overnight in the presence of 20 or 50 μg ml^−1^ PAF. Untreated samples were used as a control. The distribution of actin was determined with a Zeiss Axioplan fluorescence microscope (excitation filter 488 nm, emission filter 530 nm) as described above.

### RNA isolation and Northern blot analysis

Total RNA was isolated from *A. nidulans* mycelium treated with 50 μg ml^−1^ PAF or without PAF for 90 or 180 min, respectively, by the use of TRI Reagent (Sigma). Northern blot analysis was performed according to Kroczek and Siebert (1990). Ten micrograms of total RNA was electrophoresed on 1.2% agarose-2.2 M formaldehyde gels and blotted onto Hybond N membranes (Amersham). Digoxigenin-dUTP (Roche) labelled hybridization probes were generated by PCR using gene specific oligonucleotides as listed in Table S1. The *chsB*- and *chsD*-specific probes were generated by amplification of the corresponding gene fragments that had been cloned into the pGEM-T vector (Promega) by using the standard primers SP6 and T7. Hybridized probes were detected with anti-DIG-dUTP antibodies conjugated to horse-radish peroxidase according to the manufacturer's instructions. The quantification of band intensities on Northern blots was calculated and compared using the Image Quant Software (Molecular Dynamics).

### Analysis of the induction of the *agsA* expression by a GFP-based reporter system

The *A. niger* reporter strain RD6.47 carries the *agsA* promoter fused to a nucleus-targeted GFP (H2B::eGFP) (Meyer *et al.*, 2007). Activation of the CWI pathway can be monitored by the increase in nuclear fluorescence. Analysis of the activation of the *agsA* promoter by 50–100 μg ml^−1^ PAF was performed as described in Hagen *et al.* (2007). As a positive control, caspofungin at a concentration of 10 μg ml^−1^ was used. Fluorescence images were taken from coverslips observed with an Axioplan 2 microscope (Zeiss) equipped with a Sony DKC-5000 digital camera.

### Immunoblot analysis

Total proteins were extracted from 16–18 h cultures of *A. nidulans*, which were exposed to 50 μg ml^−1^ PAF for 30 or 90 min, respectively, or to 20 μg ml^−1^ CFW for 90 min. Controls were treated with buffer for the same duration. Mycelia were then harvested, ground in liquid nitrogen and proteins were extracted as described (Pandey *et al.*, 2004). Total protein concentration was determined using Bradford reagent. Denatured proteins (5–20 μg lane^−1^) were separated by SDS-PAGE (10% gels) and transferred to Hybond nitrocellulose membrane (Amersham), using a Tris buffer system according to Pandey *et al.* (2004). Membranes were then probed with the rabbit polyclonal antip44/42-Mpk (ERK 9101) antibodies and antiphospho p44/42 Mpk (pERK 9102) antibodies (Cell Signaling Technology) respectively. Primary antibodies were detected using anti-rabbit-α IgG–HRP conjugated secondary antibody (GE Healthcare). The antibody complex was visualized with the ECL detection system (GE Healthcare). For loading control, either membranes were stained with Ponceau solution (Sigma) after detection or control gels were Coomassie stained after SDS-PAGE.

### Detection of cAMP-dependent protein kinase

cAMP-dependent PkaA activation was determined by the use of the non-radioactive Pep Tag assay from Promega. This assay kit uses a brightly coloured, fluorescent peptide substrate, PepTag A1 peptide, that is highly specific for PKA. Phosphorylation by PKA of its specific substrate alters the peptide's net charge, which results in a separation of the phosphorylated and non-phosphorylated substrate on an agarose gel. Crude cell extracts used for the assay were extracted from 16 h cultures of *A. nidulans* FGSC4A, which had been exposed to 100 μg ml^−1^ PAF for 90 min. Controls were treated with buffer for the same duration. Mycelia were then harvested, ground in liquid nitrogen and proteins were extracted as described (Pandey *et al.*, 2004), using the PKA extraction buffer suggested in the assay protocol of Promega. Total protein concentrations were determined using the Peqlab Nanodrop. Standard cAMP-dependent protein kinase assays were carried out according to the manufacturer's instructions, using 50 μg of total protein in each sample. Controls were prepared as described in the protocol of the kit. Samples were loaded on a 0.8% agarose gel and separated at 100 V for 20 min.
