# Supplementary material for: The antifungal protein PAF interferes with PKC/MPK and cAMP/PKA signalling of Aspergillus nidulans
Source: Mol Microbiol. 2009 Nov 19;75(2):294–307. doi: 10.1111/j.1365-2958.2009.06936.x (PMC2814085; doi:10.1111/j.1365-2958.2009.06936.x)
Supplement: Supplementary file 1 [file mmi0075-0294-SD1.pdf]

SUPPLEMENTAL DATA

TABLE S1. Gene-specific oligonucleotides used for the generation of DIG-dUTP-labeled hybridisation probes.

primer	sequence	gene	protein
**AncsmAF**	5´-CCACAGACTTGAGCGTAG-3´	*csmA*	chitinsynthase
**AncsmAR**	5´-CCGTATCCATTCCCAGAG-3´		
**AnchiAF**	5´-CCTGTATGACCTTGACCC-3´	*chiA*	chitinase A
**AnchiAR**	5´-GTTGAGGTTTCCGTGTGG-3´		
**AnchsAF**	5´-GTAAGACCACCATCAGCG-3´	*chsA*	chitin synthase A
**AnchsAR**	5´-CGATACCTTTCTCAGCCC-3´		
**AnGs1F**	5´-ATCATCCCTCTCCTGTCC-3´	*agsB*	α-1,3-glucan synthase B
**AnGs1R**	5´-GCTTGCCGTACTTCTTCG-3´		
**Anb1,3GsF**	5´-GGACGAACGGTACAACAG-3´	*fksA*	β-1,3-glucan synthase A
**Anb1,3GsR**	5´-GCAGTGGGAATGTCAACG-3´		
**orlmAnF**	5'-ATCCACAACAGCATCACCC-3'	*rlmA*	ortholog of the MADS-box
**orlmAnR**	5'-ACAGCCTTATTTCCCGCAG-3'		transcription factor RlmA of *A. niger*

Figure S1

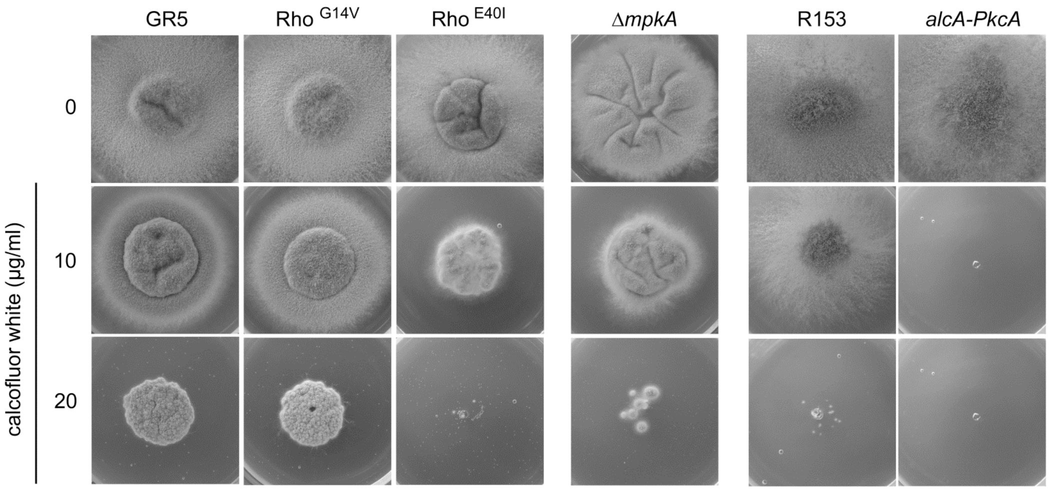

Fig. S1: Effect of CFW on *A. nidulans* strains defective in the activation of the components of the CWI pathway, (*rhoA*^{G14R}, *rhoA*^{E40I}, Δ*mpkA* and *alcA*-PkcA*)* in comparison to the respective recipient strains (GR5 for the *rhoA*^{G14R}, *rhoA*^{E40I}, Δ*mpkA* mutant strains, R153 for the conditional mutant *alcA*-PkcA). 2x10^3 conidia were point inoculated on appropriately supplemented agar plates containing 0-20 µg/ml CFW. Plates were incubated at 37°C for 48 h (RhoA and MpkA mutant strains) or for 72 h (PkcA mutant strain), respectively.

Figure S2

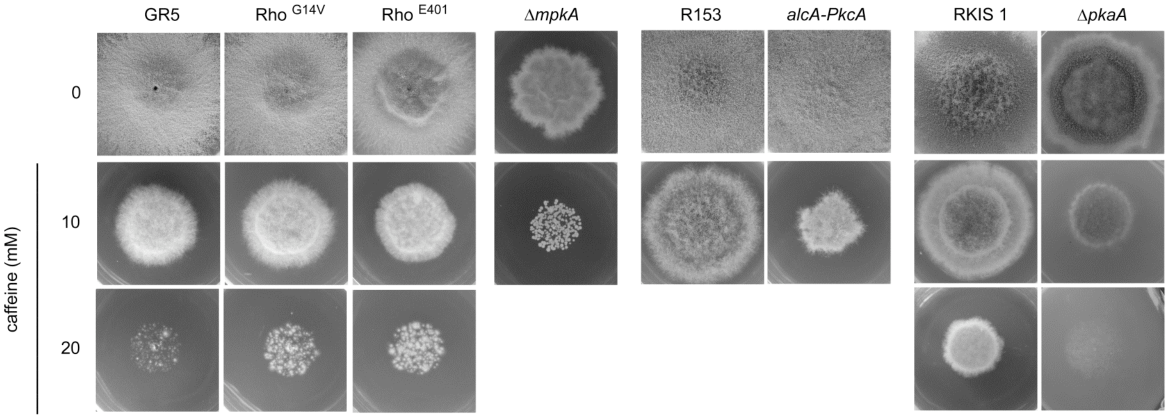

Fig. S2: Effect of caffeine on the growth of RhoA mutants, Δ*mpkA*, *alcA*-PkcA and Δ*pkaA* compared to the respective recipient strains (GR5 for both RhoA mutants and Δ*mpkA*, R153 for *alcA*-PkcA and RKIS1 for Δ*pkaA*) on solid medium. 2x10^3 conidia were point inoculated on CM containing the appropriate supplements, except for the *alcA*-PkcA mutant and its respective control strain which were grown on repressive minimal medium (MM) containing 1% glucose (Ronen *et al.*, 2007). Caffeine concentrations of 0-20 mM were tested. The *alcA*-PkcA mutant and R153 were incubated for 72 h, all other strains for 48 h.

Figure S3

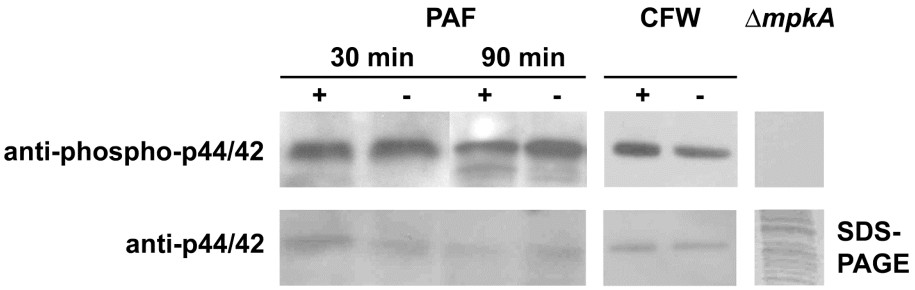

Fig. S3: Immunoblotting of *A. nidulans* cellular extracts with polyclonal anti-phospho p44/42 Mpk antibodies (anti-phospho-p44/42, upper panels). The lower panels show the results of immunoblotting of the PAF and CFW-treated samples with polyclonal anti-Mpk antibodies (anti-p44/42) as a loading control. Total proteins were extracted from *A. nidulans* without treatment (-), or after exposure to 50 µg/ml PAF (+) for 30 min and 90 min, respectively, or to 20 µg/ml CFW (+) for 90 min. Cellular extract from the *ΔmpkA* mutant strain was used to prove anti-phospho-p42/44 antibody specificity (upper panel). Coomassie-stained cellular extract of the *ΔmpkA* mutant after SDS-PAGE is shown as loading control (lower panel). 10-20 µg protein extract was loaded per lane on a 10% SDS-polyacrylamide gel.

Figure S4

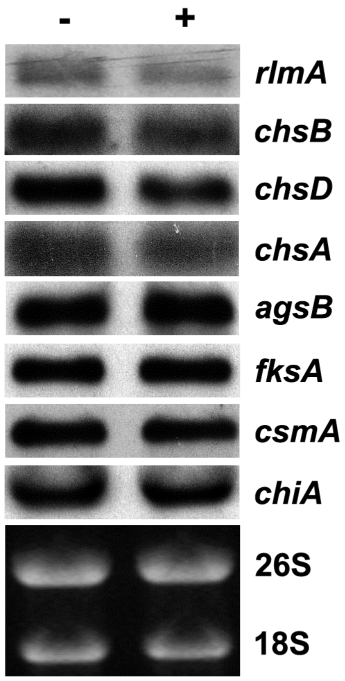

Fig. S4: Northern blot analysis of genes involved in cell wall biogenesis and remodelling. Sixteen hours old *A. nidulans* cultures were exposed to 0 (-) and 50 µg/ml PAF (+) for 3 h. Ten µg total RNA was loaded per lane and gene expression was detected by hybridizing the blotted samples with the respective DIG-dUTP-labeled probe. The lowest panel shows the 26S and 18S rRNA stained with ethidium bromide as loading control.

Figure S5

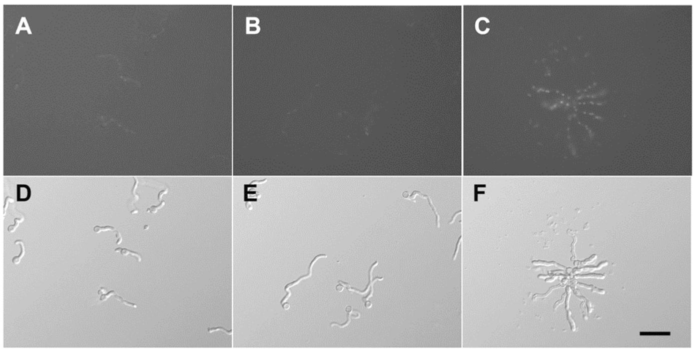

Fig. S5: Microscopic analysis of *A. niger* strain RD6.47 expressing a nucleus-targeted GFP under the control of the *A. niger agsA* promoter. Five hours old germlings were treated with 100 µg/ml PAF **(B, E)** or 10 µg/ml caspofungin **(C, F)** as a positive control for additional 2 h. Untreated germlings served as negative control **(A, D)**. Nuclear staining was monitored by fluorescence microscopy **(A-C)** as described in Materials and Methods. **(D-F)** represent differential interference contrast images of the respective germlings. Scale bar, 20 µm.

Figure S6

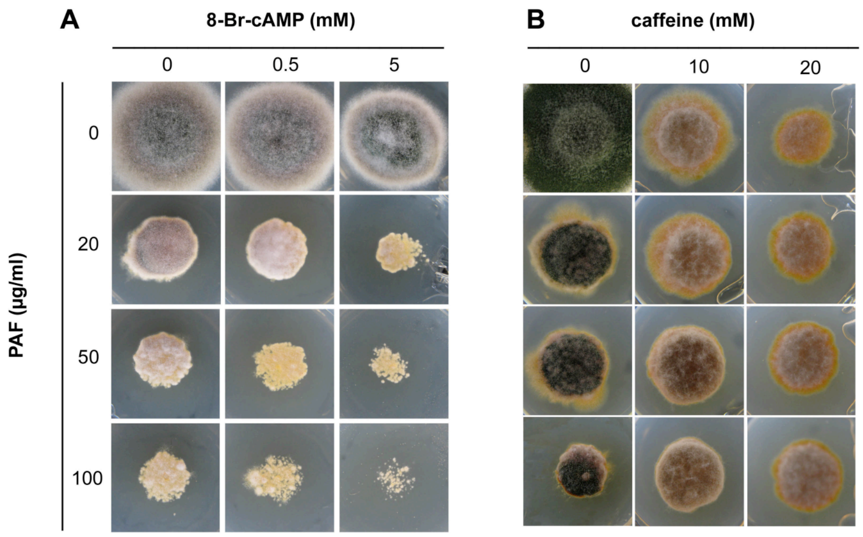

Fig. S6: Effect of the cAMP/PkaA signaling pathway modulating substances 8-Br-cAMP **(A)** and caffeine **(B)** in combination with PAF on the growth of *A. nidulans* FGSC4A. 2x10^3 conidia were point inoculated on CM agar plates containing 0-100 µg/ml PAF combined with 0-5 mM 8-Br-cAMP or 0-20 mM caffeine. Plates were incubated at 37°C for 48 h in **(A)** and for 72 h in **(B)**.

Figure S7

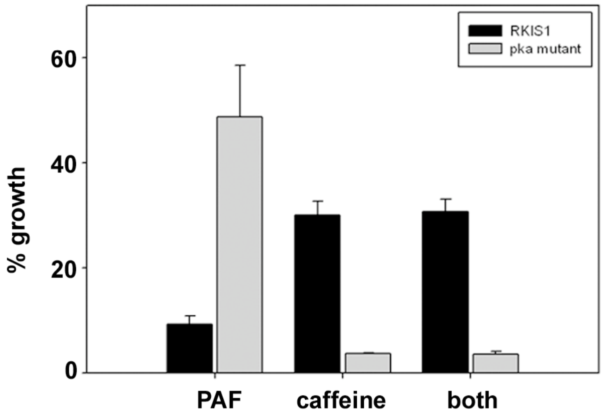

Fig. S7: Liquid growth inhibition assay of *A. nidulans* Δ*pkaA* and *RKIS1* exposed to PAF (5 µg/ml), caffeine (10 mM) or to a combination of both substances. The % growth was calculated from % changes in OD_{620} of compound-treated conidia compared to the untreated control (=100%). Note that for growth assays in liquid culture (96-well plates) less concentration of growth inhibitory compounds is needed compared to agar plate assays.
